# Cross-cultural adaptation of the Skin Cancer Index into Brazilian Portuguese for patients with cervicofacial nonmelanoma skin cancer

**DOI:** 10.1007/s00520-023-08051-4

**Published:** 2023-09-23

**Authors:** Evânia Curvelo Hora, Marcela Sampaio Lima, Hianga Fayssa Fernandes Siqueira, Adriane Dórea Marques, Érika de Abreu Costa Brito, Arthur Leite Lessa, Carlos Anselmo Lima

**Affiliations:** 1 Emergency Hospital of Sergipe (Hospital de Urgências de Sergipe), Aracaju, Sergipe, Brazil; 2 Health Sciences Graduate Program, Federal University of Sergipe (Universidade Federal de Sergipe), Aracaju, Sergipe, Brazil; 3 University Hospital (Hospital Universitário), Federal University of Sergipe (Universidade Federal de Sergipe), Aracaju, Sergipe, Brazil; 4 Department of Medicine, Federal University of Sergipe (Lagarto Campus), Lagarto, Sergipe, Brazil; 5 Ministry of Health (Ministério da Saúde-MS), Aracaju, Sergipe, Brazil; 6 Department of Medicine, Federal University of Sergipe (Universidade Federal de Sergipe), Aracaju, Sergipe, Brazil; 7 School of Medicine, Federal University of Sergipe (Universidade Federal de Sergipe), Aracaju, Sergipe, Brazil; 8 State Department of Health (Secretaria de Estado da Saúde), Aracaju, Sergipe, Brazil

**Keywords:** Cross-cultural adaptation model, Nonmelanoma skin cancer, Portuguese, Skin Cancer Index

## Abstract

**Purpose:**

This study aimed to translate the Skin Cancer Index (SCI) into Portuguese, adapt it for Brazilian culture, and clinically validate it.

**Methods:**

A five-stage cross-cultural adaptation model was followed, with subsequent clinical validation. Inter-rater agreement was assessed using the content validity index (CVI). The hypothesis of the non-inferiority of the CVI at 80% probability level was evaluated using an exact binomial test. We used Spearman’s rank-order and Pearson’s product–moment correlation analysis, internal consistency using McDonald’s *ω* and Cronbach’s *α* metric, and construct validity using confirmatory factor analysis. The factorial model was validated using the chi-squared test, root mean square error of approximation (RMSEA), comparative fit index (CFI), and standardized root mean square residual (SRMR).

**Results:**

The first stage yielded two independent translations. After synthesis, back-translation, and review, the prefinal version was tested on 40 patients. Inter-rater agreement indices on content validity were significantly higher than 80% (*p* < 0.05). The SCI remained stable, and the Spearman’s rank-order (*r*_*s*_), Pearson product–moment (*r*), and intraclass correlation coefficients were > 0.9, indicating excellent reliability. The reliability of McDonald’s *ω* was considered ideal (> 0.8) in all subdimensions and scale. Cronbach’s *α* was considered ideal in the “Emotional” and “Social” subdimensions and scale. Construct validity was observed in all subdimensions and scale through the criteria (*χ*^2^) *p* value > 0.05, RMSEA < 0.08, CFI ≥ 0.9, and SRMR ≤ 0.08.

**Conclusion:**

The cross-cultural adaptation of the SCI to Portuguese for Brazilian culture showed content validity and reliability, contributing to quality of life assessment in patients with NMSC.

## Introduction

Nonmelanoma skin cancer (NMSC), the malignant neoplasm with the highest incidence worldwide, accounts for approximately 30% of all malignant tumors recorded in Brazil. Estimates indicate that 83,770 new cases are diagnosed annually among men and 93,160 among women in Brazil [1]. Therefore, NMSCs are a major public health problem with strong economic and social impact [2]. Approximately 95% of NMSCs consist of basal and squamous cell carcinomas, and 70% of NMSCs occur in the cervicofacial region due to the increased exposure of these areas to ultraviolet radiation, particularly from ultraviolet B rays [2–4].

The gold standard treatment for NMSCs is surgical resection, which has a low mortality rate; however, several studies [3–11] have demonstrated that the diagnosis and treatment of this disease cause high morbidity due to its various symptoms and physical, emotional, and social sequelae, with high levels of anguish, anxiety, depression, and social isolation. These effects impact the patient’s general health and, therefore, impair their quality of life.

Health-related quality of life is the perception of disease and treatment effects on the physical, psychological, and social aspects of a patient’s life [6, 8, 11]. Instruments that assess quality of life may be generic or specific: the former may not accurately identify the quality of life of a patient with a particular disease because some items may lack relevance, whereas the latter may be applied to a specific group and therefore be more sensitive in assessing a specific disease [6, 11].

In 2005, Rhee et al. created the first specific instrument in English for assessing the quality of life of patients with cervicofacial NMSC, termed the Skin Cancer Index (SCI) [12–15]. In a recent systematic review, the SCI was identified as the only easily applied specific instrument developed and validated to measure the quality of life of patients with cervicofacial NMSC [7, 10, 15].

The SCI has been shown to be sensitive and responsive in assessing the quality of life of patients with cervicofacial NMSC. In addition, it enables future comparative studies aimed at assessing the impact of NMSC on quality of life by subgroup as a function of, among others, treatment, sex, ethnicity, region, location, and severity of lesions [14]. A Spanish version of the SCI was adapted and validated to the Spanish language and the culture by Troya-Martín et al. [16, 17].

In this context, the objective of this study was to cross-culturally adapt the SCI to Portuguese and the Brazilian culture, as well as clinically validate the adaptation. The intention of this objective was to provide the scientific community with a valid and specific instrument for clinical studies in patients with cervicofacial NMSC, and thereby help to understand the impact of this disease on patients’ quality of life.

## Materials and methods

The study comprised two stages. The first stage consisted of methodological research on the cross-cultural adaptation of the SCI and evaluation of the psychometric properties required for the Brazilian version. The second stage was a cross-sectional clinical validation study using 182 patients.

The SCI consists of 15 items divided into three subscales to evaluate the quality of life of patients with cervicofacial NMSC: emotional (7 items), social (5 items), and appearance (3 items). Each item is scored from 1 to 5 (1 = extremely, 2 = very much, 3 = moderately, 4 = slightly, 5 = not at all), with a total score ranging from 0 to 100.

The original version of the SCI was created in 2005 by Rhee et al. in the USA [12]. Therefore, authorization to conduct the cross-cultural adaptation of the SCI scale to northeastern Brazil was requested from and granted by the authors. This project was approved by the Human Research Ethics Committee of the Federal University of Sergipe (*Universidade Federal de Sergipe*), Brazil, under CAAE (Certificado de Apresentação de Apreciação Ética) number 02241518.5.0000.5546/Opinion: 3.011.994. The study was performed in accordance with the ethical standards as laid down in the 1964 Declaration of Helsinki and its later amendments or comparable ethical standards. The participants’ identity and rights were preserved in compliance with Resolution Number 466, of December 12, 2012, of the National Health Council (*Conselho Nacional de Saúde*) of the Ministry of Health (*Ministério de Saúde*), Brasília – Federal District (Distrito Federal) of Brazil.

Using a questionnaire in another country, culture, or language requires the development of its own method to achieve equivalence between the original (where it was developed) and intended (where it will be used) versions. Items must not only be well translated linguistically, but also culturally adapted to maintain the content validity of the instrument in different cultures [18–20]. In the first stage, the study followed the method proposed by Beaton et al. [19] with the following five steps.

### Step I—Initial translation

The SCI was directly translated from English (source language) to Portuguese (target language) by two independent bilingual translators from Brazil, whose mother tongue is Portuguese. This step resulted in two translations, T1 and T2, and was followed so that the translations could be compared and any differences could be identified and analyzed.

### Step II—Synthesis of the translations

A meeting was held between the translators and researchers to synthesize translations T1 and T2. During the meeting, discrepancies between the two translations were discussed, as well as the adequacy of the instrument for different educational levels and age groups. A single consensus translation (T1-2) resulted.

### Step III—Back translation

During this stage, inconsistencies, differences, and conceptual errors in T1-2 were highlighted to ensure that the translated version accurately reflected the content of the original version. T1-2 was back translated (BT1, BT2) into English by two independent American translators who are native English speakers, albeit fluent in Portuguese.

### Step IV—Expert committee review

A committee of experts reviewed the versions and components of the scale to strengthen the prefinal version of the instrument. The committee searched for semantic, idiomatic, cultural, and conceptual equivalence between the original instrument and its consensus-translated version.

The committee consisted of healthcare professionals with clinical experience of 10 years or longer in treating patients with cervicofacial NMSC and conducting validation studies. In total, 12 healthcare professionals participated in this study: two each of oncologists, plastic surgeons, head and neck surgeons, dermatologists, psychologists, and nurses. All experts rated the suitability of the Brazilian version of the SCI for its purpose using the “Guidelines for the Process of Cross-Cultural Adaptation of Self-Report Measures [19]”.

### Step V—Testing of the prefinal version

Testing the prefinal version was the final stage of the adaptation process. This pilot cross-sectional prospective study included 40 patients with cervicofacial NMSC. Once demographic and clinical data were collected, the prefinal version of the SCI adaptation (test) was applied. After 1 week, the prefinal version of the SCI (retest) was applied again. This stage was important to assess whether the meaning and answers confirmed that the adapted version retained the measurement properties required to fulfill its purpose–that is, its equivalence in the intended application.

The second stage was a cross-sectional study for clinical validation using a sample of 182 patients with cervicofacial NMSC treated in public hospitals (University Hospital [*Hospital Universitário*], Federal University of Sergipe [*Universidade Federal de Sergipe*], São Cristóvão and Lagarto campus, and Oncology Center [*Centro de Oncologia*]), the Emergency Hospital of Sergipe (*Hospital de Urgência de Sergipe*), and a private clinic in Sergipe, Brazil.

Patients were recruited during their first visit, during which they were examined by a plastic surgeon. The study inclusion criteria were as follows: patients from Brazil, older than 18 years, with a diagnosis of cervicofacial NMSC and a therapeutic plan for surgical resection of the lesion, whose cognitive conditions and verbal expressions enabled them to participate when the assessment instruments were applied, and who agreed to participate in the study voluntarily. Patients with neuropsychiatric disorders that prevented them from understanding and completing the questionnaire were excluded.

After consenting to participate in the study by completing an informed consent form (*Termo de Consentimento Livre e Esclarecido*), the patients were interviewed in the preoperative period (T0), when the demographic data were first collected. The data included address, place of birth, age, ethnicity, religion, marital status, education, profession, and family income. The clinical variables included the type, duration, number, size, and location of the lesions as well as symptoms associated with the lesions, prior surgeries for other lesions, H-zone involvement, functional involvement, first intervention or reoperation, margin expansion or lesion recurrence, and diagnostic hypothesis and therapeutic plan (primary wound closure, grafts, or flaps).

Once demographic and clinical data were collected, the final version of the SCI adaptation (T0) was applied. The patients underwent surgery, and their diagnosis of NMSC was confirmed with an anatomopathological examination. The final version of the SCI was applied in the postoperative period (T1) 4 months after the surgery.

Because the authors participated in the data collection, they had access to information that could identify individual participants during and after the data collection.

### Statistical analysis

In the first stage, inter-rater agreement was assessed using the content validity index (CVI). The hypothesis of the non-inferiority of CVI at the 80% probability level was evaluated using an exact binomial test. The absence of agreement between the measurements was tested using the intraclass correlation coefficient (ICC) and its 95% confidence interval. The absence of a correlation between the measures was tested using Spearman’s rank-order and Pearson’s product–moment correlation analysis.

Categorical variables were described using absolute and relative percentage frequencies, and continuous variables were described as means (with standard deviations) and medians (with interquartile ranges) in both stages.

In the second stage, to assess internal consistency, McDonald’s *ω* [21] and Cronbach’s *α* metrics were used; a value greater than 0.8 is considered ideal and thus an indicator of instrument quality. To assess construct validity, confirmatory factor analysis was applied. The validity of the factorial model was confirmed using the chi-squared test, root mean square error of approximation (RMSEA), comparative fit index (CFI), and standardized root mean square residual (SRMR). The objective was to meet the following adjustment criteria: (*χ*^2^) *p* value > 0.05; RMSEA < 0.08; CFI ≥ 0.9; SRMR ≤ 0.08 [22, 23].

The significance level was set to 5%, and the software used for the analysis was R Core Team (2022; version 4.2.0) [24].

## Results

The three initial stages of the cross-cultural adaptation were completed using a version of the SCI that was evaluated by experts. Table 1 presents the inter-rater agreement indices for content validity, whose values were significantly (*p* < 0.05) higher than 80% based on the exact binomial test, as recommended for content validation.

**Table 1:** Percentage inter-rater agreement indices according to the translated version of the Skin Cancer Index equivalence types. Exact binomial test**,** Sergipe, Brazil, 2020

		Equivalence							
		Semantic		Linguistic		Experimental		Conceptual	
Item	Sentence/translation	CVI	*p*	CVI	*p*	CVI	*p*	CVI	*p*
1	A – Are you worried that the skin cancer might spread to another part of your body? B – Tem se preocupado com a possibilidade de o seu câncer de pele se espalhar para outras partes do corpo?	100%	1.000	100%	1.000	100%	1.000	100%	1.000
2	A – Do you feel anxious about your skin cancer? B – Tem se sentido ansioso (a) por causa do câncer de pele?	100%	1.000	100%	1.000	100%	1.000	100%	1.000
3	A – Are you worried that family members may also develop skin cancer? B – Tem se preocupado com a possibilidade de outros membros da família também desenvolverem o câncer de pele?	100%	1.000	100%	1.000	100%	1.000	100%	1.000
4	A – Are you worried about the cause of skin cancer? B – Tem se preocupado com a causa do câncer de pele?	100%	1.000	100%	1.000	100%	1.000	100%	1.000
5	A – Do you feel frustrated about your skin cancer? B – Tem se sentido frustrado (a) com seu câncer de pele?	100%	1.000	100%	1.000	100%	1.000	100%	1.000
6	A – Are you worried that your tumor may become a more serious type of skin cancer? B – Tem se preocupado se o seu tumor pode se tornar um tipo mais grave de câncer de pele?	100%	1.000	100%	1.000	100%	1.000	100%	1.000
7	A – Are you worried about new skin cancer occurring in the future? B – Tem se preocupado com o surgimento de novos cânceres de pele no futuro?	100%	1.000	100%	1.000	92%	0.931	100%	1.000
8	A – Felt uncomfortable when meeting new people? B – Tem se sentido desconfortável ao conhecer novas pessoas?	100%	1.000	100%	1.000	100%	1.000	100%	1.000
9	A – Felt concerned that your skin cancer may worry friends or family? B – Tem se sentido apreensivo (a) se seu câncer de pele pode preocupar amigos e familiares?	92%	0.931	100%	1.000	100%	1.000	100%	1.000
10	A – Worried about the length of time before you can go out in public? B – Tem se preocupado em quanto tempo poderá sair em público?	100%	1.000	100%	1.000	100%	1.000	100%	1.000
11	A – Felt bothered by people’s questions related to your skin cancer? B – Tem se incomodado com as perguntas das pessoas sobre o seu câncer de pele?	100%	1.000	100%	1.000	100%	1.000	100%	1.000
12	A – Felt embarrassed by your skin cancer? B – Tem se sentido envergonhado (a) com seu câncer de pele?	100%	1.000	100%	1.000	100%	1.000	100%	1.000
13	A – Worried about how large the scar will be? B – Tem se preocupado com o tamanho da cicatriz?	100%	1.000	100%	1.000	100%	1.000	100%	1.000
14	A – Thought about how skin cancer affects your attractiveness? B – Tem pensado como o câncer de pele afeta sua aparência?	100%	1.000	100%	1.000	100%	1.000	100%	1.000
15	A – Thought about how noticeable the scar will be to others? B – Tem pensado o quanto as pessoas irão perceber sua cicatriz?	100%	1.000	100%	1.000	100%	1.000	100%	1.000

*CVI*, content validity index

During the test phase of the prefinal version, all 40 patients recruited for the study answered the questionnaire at two time points (test and retest) with 7 days. They then underwent classic excision of the lesion, and after 10–15 days, anatomical pathology confirmed the suspected diagnosis of NMSC.

The approximate time for completing the questionnaire was between 5 and 10 min, and no patients refused to complete the questionnaire. There was no report of misunderstanding the items, nor was there any lack of response to any question. Illiterate patients were assisted by their companions, who read the questions to them.

The demographic characteristics of the patients included a male–female parity, mean age of 65.7 ± 11.7 years, and mean time from symptom onset of 300.1 ± 549.7 days. Most patients (94.9%) had children (on average, 3.5 ± 2.6 children), had more than 10 years of schooling (57.5%), were white (87.5%), and were married (79.5%).

The clinical characteristics showed a higher proportion of single lesions with an average size of 1.44 cm ± 1.43 cm, predominantly located in the H-zone, especially on the nose, and the main symptom was lesion growth. Most patients presented with high blood pressure (57.5%) and diabetes (35%) as comorbidities, and 32.5% had a history of other cervicofacial NMSCs. The prevailing surgical treatment was a skin flap (65%), and histopathological results confirmed basal cell carcinoma in 85% of cases, of which 32.4% were solid ulcerated lesions.

Reliability was assessed using the ICC, Spearman’s rank-order (*r*_*s*_), and Pearson product–moment (*r*) correlation analyses between the test and retest, as presented in Table 2. No significant differences were observed between the SCI test, retest, and its dimensions (*p* < 0.05); hence, the means of the two evaluations were similar. In addition, Spearman’s rank-order and Pearson product–moment correlation coefficients and ICC were all significantly higher than 0.9, indicating excellent reliability. Thus, the SCI remained stable over the 7-day study period.

**Table 2:** Descriptive statistics (assessed by intraclass correlation coefficient, Spearman’s rank-order (*rs*), and Pearson’s product–moment (*r*) correlation analysis) of the adapted Skin Cancer Index subscales between the test and retest, Sergipe, Brazil, 2020

	Test	Retest	Difference mean	ICC (95% CI)	Pearson’s (*p* value)	Spearman’s (*p* value)
	Mean (SD)	Mean (SD)				
SCI	78.5 (19.9)	78.4 (19.7)	0.132	0.990 (0.984–0.994)	0.991 (< 0.001)	0.972 (< 0.001)
Emotional	72.5 (23.1)	72.5 (21.9)	0.005	0.979 (0.964–0.988)	0.980 (< 0.001)	0.964 (< 0.001)
Social	86.5 (20.7)	86.6 (19.9)	− 0.125	0.989 (0.970–0.994)	0.989 (< 0.001)	0.947 (< 0.001)
Appearance	79.2 (24.9)	78.3 (25.2)	0.830	0.972 (0.941–0.991)	0.972 (< 0.001)	0.986 (< 0.001)

*SCI*, Skin Cancer Index; *SD*, standard deviation; *ICC*, intraclass correlation coefficient; *95% CI*, 95% confidence interval

*p* < 0.05

In the second phase of the study, nine of the 182 patients who responded to T0 had their follow-up interrupted due to loss of contact at T1 when the questionnaire was administered, but this did not compromise the statistical viability.

Table 3 presents the descriptive and psychometric SCI statistics. The averages of the “Emotional” dimension varied from 3.538 to 4.258, “Social” dimension varied from 4.093 to 4.659, and “Appearance” dimension from 4.156 to 4.280. McDonald’s *ω* reliability was considered ideal (> 0.8) in all subdimensions and in the scale. Cronbach’s *α* was considered ideal in the “Emotional” and “Social” subdimensions in addition to the scale. Reliability was guaranteed in the subdimensions and in the scale.

**Table 3:** Descriptive and psychometric statistics from the adapted Skin Cancer Index, Sergipe, Brazil, 2021–2022

	Average	SD	McDonald’s *ω*	Cronbach’s *α*	${\chi }^{2}$ (*p*value)	RMSEA	CFI	SRMR
Emotional			0.917	0.895	7.26 (0.924)	0.000	1.000	0.041
SCI1	3.797	1.243						
SCI2	3.819	1.228						
SCI3	3.852	1.219						
SCI4	3.945	1.179						
SCI5	4.258	1.100						
SCI6	3.538	1.307						
SCI7	3.654	1.238						
Social			0.834	0.753	2.97 (0.709)	0.000	1.000	0.044
SCI8	4.604	0.839						
SCI9	4.093	1.091						
SCI10	4.599	0.922						
SCI11	4.659	0.901						
SCI12	4.621	0.857						
Appearance			0.897	0.830	0.000 (1.000)	0.000	1.000	0.000
SCI13	4.280	1.063						
SCI14	4.198	1.105						
SCI15	4.156	0.878						
**Total**			0.912	0.905	68.87 (0.924)	0.000	1.000	0.068

*SD*, standard deviation; *RMSEA*, root mean square error of approximation; *CFI*, comparative fit index; *SRMR*, standardized root mean square residual; *SCI*, Skin Cancer Index

In all subdimensions and in the scale, the criteria (*χ*^2^) *p* value > 0.05, RMSEA < 0.08, CFI ≥ 0.9, and SRMR ≤ 0.08 were guaranteed, indicating construct validity of the scale.

**Fig. 1:**
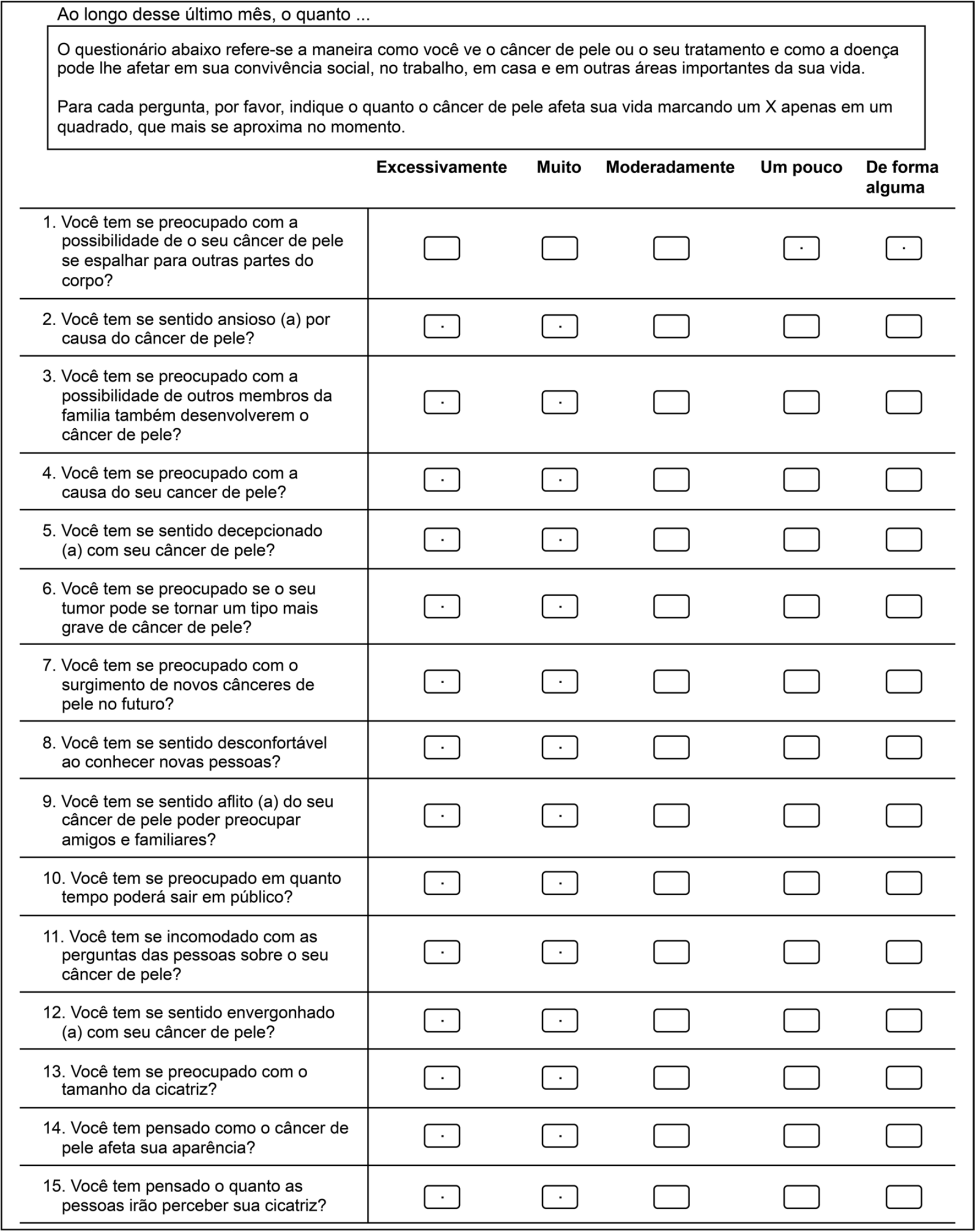
Final version of the cross-cultural adaptation of the Skin Cancer Index to Portuguese, Sergipe, Brazil, 2020

The final version of the SCI translated to Portuguese and adapted to Brazilian culture had the same number of items as the original version (Fig. 1).

## Discussion

The SCI Portuguese version adapted to Brazilian culture followed a rigorous method for the cross-cultural adaptation of instruments. All 15 items of the original scale were validated and retained in this adapted version to Portuguese, while the Spanish version reduced the scale to 13 items [16].

The feasibility of the version to assess the quality of life in patients with NMSC was initially evidenced by the ability of patients to answer the questionnaire in a short time (5–10 min), adequately understand the questions, and answer all questions. In other versions of the same instrument, the time to complete the questionnaire was even shorter (3–6 min), which reveals cultural differences between countries and even regions of the same country [16].

The inter-rater agreement indices for content validity were higher than 80%, similar to previous studies conducted using the same instrument [12, 16]. This reflects the degree to which the instrument assesses what it proposes to measure according to the chosen methodological recommendation, making this phase a crucial part of the cross-cultural adaptation process [18].

The ICC and Spearman’s and Pearson’s correlations showed values greater than 0.9, indicating high reliability of the instrument, consistent with the findings of a study of the Spanish version by Kappa [16].

By examining all subscales, both individually and in the total SCI score, the presurgical scores demonstrated an effect on the quality of life of the patients. This finding suggests that the routine use of the SCI in clinical settings can identify patients vulnerable to preoperative psychosocial concerns, rather than basing such identification on assumptions related to age and sex [8].

Regarding the psychometric properties of the adapted version of the SCI, we observed that the intra-dimension reliability measured using McDonald’s *ω* was considered ideal in all subdimensions and in total. However, using Cronbach’s *α*, it was considered ideal in the emotional, appearance, and total subdimensions, and reasonable in the social one. It is noteworthy that this last metric usually underestimates reliability and should be evaluated with caution [21]. Therefore, in the subdimensions and scale, reliability was guaranteed in accordance with other studies [12, 16].

The construct validity of the adapted version was guaranteed in all subdimensions and in the scale. We observed that all results met the adjustment criteria (*χ*^*^*2^)* p* value > 0*.*05, RMSEA < 0.08, CFI ≥ 0.9, and SRMR ≤ 0.08. Thus, we can state that the instrument measures the intended construct, in agreement with the other versions of the instrument [12, 16].

A study [5] conducted with 52 patients to measure quality of life acceptance in patients with NMSC, using the Dermatology Life Quality Index—a generic characteristic questionnaire for skin diseases—showed a good acceptance among patients who completed the questionnaire. However, the results suggested the need to consider a specific instrument for this evaluation. Unlike other neoplasms, NMSCs have not been systematically investigated regarding quality of life despite the potential importance of quality of life as an endpoint of the disease process among patients with this type of neoplasm. This is particularly due to the high morbidity and probability of developing a second primary NMSC in subsequent years. Accordingly, NMSCs can recur as an acute exacerbation of the chronic form of the disease [12].

A systematic review [11] of several instruments for assessing quality of life using the COnsensus-based Standards for the selection of health status Measurement INstruments (COSMIN) checklist and the proposed criteria of Terwee et al., identified four questionnaires in a synthesis of the best evidence, including the SCI [25]. Corroborating this study, a literature review [26] of quality of life assessment instruments in patients with NMSC and melanoma highlighted the SCI as a valid and useful instrument in several studies that evaluated quality of life in patients with NMSC.

Therefore, the SCI is considered a simple, solid, consistent, reliable, and valuable instrument, not only in clinical practice but also in epidemiological and clinical research on patients with cervicofacial NMSC [17]. However, because the SCI assesses a specific disease, a significant limiting disadvantage is that it cannot be used to compare the disease impact on quality of life across a broad spectrum of dermatological diseases [6]. In addition, the SCI fails to address the patient’s distress or occupational concerns. These issues are particularly important for younger patients, among whom the annual incidence of the disease is increasing [27].

Recent studies [28–30] used the SCI as an instrument to measure quality of life in patients with head and neck skin cancer and melanomas. This may expand the applicability of this instrument.

The increased prevalence of chronic diseases and the promotion of self-care, as well as advances in information technology, account for the increasing interest of the scientific community in patients’ opinions about changes in their quality of life when faced with a disease and the related treatment outcomes. These measurements can only be performed by asking patients. Therefore, to ensure the validity of the answers, instruments must be linguistically and culturally adapted for those who will be required to complete such questionnaires.

The present study strictly complied with the chosen methodological recommendations [19]; however, it had limitations regarding the sample. The study was conducted in only two cities (Aracaju and Lagarto) in northeastern Brazil, although many participants were from nearby regions with specific sociocultural characteristics.

Brazil is a country of continental dimensions with an estimated population of 215 million people [31] and there could therefore be greater heterogeneity in the sample, which does not invalidate the possibility that new studies could be carried out with larger and more diversified sample populations that may contribute to the results of applying the adapted version of the SCI to Portuguese.

It is noteworthy that the two collection phases (pre-test and clinical validation) were carried out during the coronavirus disease 2019 pandemic, specifically between the waves of the disease, when there was a partial opening of outpatient health services. Thus, the study was conducted at a time when the world’s population was exposed to a significant stress load resulting from the uncertainties inherent in the pandemic state; this may have negatively affected the quality of life measurement of these individuals.

Considering the above, the cross-cultural adaptation of instruments is crucial for their use in different populations and cultures to translate the studied theme as reliably as possible. In this sense, the cross-cultural adaptation of the SCI for Brazil is viable and provides another version in Portuguese, given the large territorial dimensions of Brazil. Brazil stands out as the largest country in Latin America and the sixth most populous country worldwide, with great sociocultural differences.

In conclusion, measuring the quality of life of patients with cervicofacial NMSC is a valuable resource for clinical practice and research. The publication of this adapted SCI version will allow for further clinical validation studies with larger sample sizes. In addition, it may facilitate the elaboration of SCI versions in Portuguese spoken on other continents, as well as interest researchers from countries with different languages in creating new versions. Thus, it may enable comparative evaluation studies to be conducted with the consequent elaboration of specific intervention programs that can contribute to the improvement of the quality of life of this population.
